# Serotonin-modulating therapies for the management of chronic wounds

**DOI:** 10.3389/fphar.2025.1656302

**Published:** 2025-09-22

**Authors:** Anuj Budhiraja, Alisha Mehta, Johanna Ghebrehiwet-Kuflom, Janmesh D. Patel, Christiane How-Volkman, Lara Ali, Sara Dahle, Roslyn Rivkah Isseroff

**Affiliations:** ^1^ California Northstate University College of Medicine, Elk Grove, CA, United States; ^2^ Howard University College of Medicine, Washington, DC, United States; ^3^ VA Northern California HealthCare System, Dermatology Section, Mather, CA, United States; ^4^ VA Northern California Health Care System, Podiatry Section, Mather, CA, United States; ^5^ Department of Dermatology, University of California, Davis, CA, United States

**Keywords:** wound healing, chronic wounds, serotonin, selective-serotonin reuptake inhibitor, serotonin-modulating pharmacotherapy

## Abstract

**Introduction:**

Chronic wounds are a significant source of patient morbidity, and ineffective treatment can lead to complications that are difficult and costly to manage. Given the limitations of current therapies, repurposing medications with well-studied safety and accessibility profiles offers a promising strategy for advancing wound care.

**Methods:**

A comprehensive review of the existing literature was conducted to evaluate the role of serotonin-modulating pharmacotherapy in wound healing.

**Results:**

Serotonergic signaling plays a multifaceted role in wound healing and evidence increasingly supports serotonin-modulating pharmacotherapy as having favorable angio-regulatory, immunomodulatory, and antimicrobial wound healing effects. Preclinical and clinical studies have demonstrated that topical administration of serotonin-modulating pharmacotherapy may improve wound healing outcomes.

**Discussion:**

findings of this study provide support for the use of serotonin-modulating pharmacotherapy, with a special focus on topical application, as an adjunctive treatment for chronic, non-healing wounds and highlight the need for further translational clinical investigation.

## 1 Introduction

Chronic non-healing wounds, such as neuropathic and vascular ulcers, cause significant disability and increase the risk of pain, infection, sepsis, amputation, and other morbidities (Zhao et al., 2016). In the United States, chronic wounds affect nearly 2.5% of the population and contribute to an economic burden surpassing $50 billion annually (Sen, 2019; Sen, 2021). Serotonin also known as 5-hyrdoxytryptamine (5-HT) is an extensively studied monoamine neurotransmitter that is also synthesized and used by peripheral cells (Malinin et al., 2004; Alstergren et al., 1999; Laberge et al., 1996; Nguyen et al., 2019). Tryptophan hydroxylase 1, a key peripheral serotonin-synthesizing enzyme, is expressed in lymphocytes, macrophages, mast cells, and T cells, while serotonin transporter (SERT) and 5-HT receptors are also present in macrophages, dendritic cells, and lymphocytes (Shah and Amini-Nik, 2017). In response to peripheral inflammation, both 5-HT and its receptors are upregulated, promoting key angiogenic and cellular wound healing pathways (Malinin et al., 2004; Alstergren et al., 1999; Laberge et al., 1996; Nguyen et al., 2019). At present, 5-HT is underappreciated in the wound healing literature, but its developing multifaceted involvement positions 5-HT signaling as a promising target for advanced wound care therapeutics.

## 2 Mechanistic basis and therapeutic rational for Serotonin/SSRIs in wound healing

### 2.1 Overview and clinical framing

**FIGURE 1:**
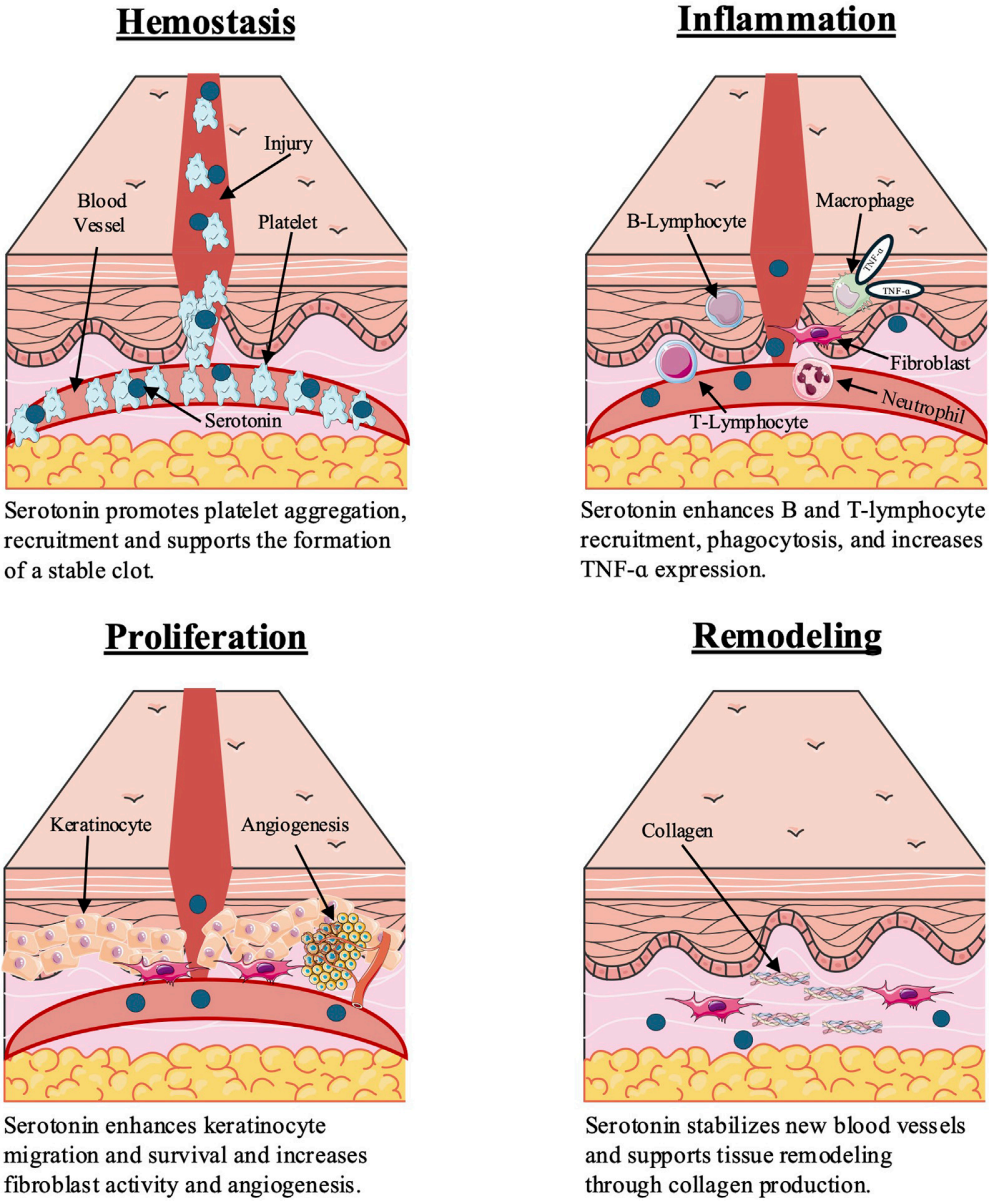
Serotonin influences wound healing stages through effects on platelets, immune cells, fibroblasts, and keratinocytes. Figure created by the authors based on findings from Enoch and Leaper (2008), Opneja et al. (2019), and Shah and Amini-Nik (2017).

5-HT plays regulatory roles in the four progressive, overlapping stages of wound healing: hemostasis, inflammation, proliferation, and remodeling (Figure 1) (Enoch and Leaper, 2008; Eskeland et al., 2017). Selective serotonin reuptake inhibitors (SSRIs), including fluoxetine, citalopram, escitalopram, sertraline, and paroxetine, inhibit 5-HT reuptake at the synaptic cleft and are increasingly used across a broadening range of clinical applications including dermatologic diseases such as atopic dermatitis, contact dermatitis, and psoriasis, partially due to their well-documented downregulation of various proinflammatory cytokine signatures (Brody and Gu, 2020; Eskeland et al., 2017; Kiecka and Szczepanik, 2022; Shah and Amini-Nik, 2017). Emerging evidence supports the use of SSRIs to improve wound healing outcomes via angio-regulatory, immunomodulatory, and antimicrobial mechanisms, which remain under-characterized and have not yet been comprehensively contextualized for cutaneous wound healing (Eskeland et al., 2017).

### 2.2 Hemostasis and angiogenesis

Within minutes of cutaneous injury in the hemostasis stage, thrombin triggers 5-HT release from platelets and endothelial cells, activating 5-HT receptors on the same cell types and initiating G-protein–mediated extracellular signal–regulated kinases 1 and 2 phosphorylation in the mitogen-activated protein kinase signaling pathway (Iwabayashi et al., 2012; Raote et al., 2007; Machida et al., 2013; Qin et al., 2013; Olszewska-Pazdrak and Carney, 2013; Gonzalez de Valdivia et al., 2017; Guo et al., 2022; Flaumenhaft and De Ceunynck, 2017; Huang et al., 2015; Tsopanoglou and Maragoudakis, 1999; Duerschmied et al., 2013). This early 5-HT autocrine signaling amplifies downstream pro-angiogenic cascades by upregulating vascular endothelial growth factor (VEGF) receptors and promoting release of VEGF, CXCL12, and matrix metalloproteinases, to sustain VEGF and nitric oxide–driven neovascularization (Almalki and Agrawal, 2017; Lin et al., 2023; Olszewska-Pazdrak and Carney, 2013; Guo et al., 2022; Flaumenhaft and De Ceunynck, 2017; Yang et al., 2005; Ceradini et al., 2004). SSRIs may be leveraged to attenuate dysregulated 5-HT signaling and to modulate the aberrant platelet and vascular responses in pathologic chronic wounds. For example, systemic SSRIs block SERT on platelets, preventing 5-HT uptake and causing a dose-dependent decrease, often exceeding 80%, in platelet 5-HT with greater effects seen after 6–12 weeks (Maguire et al., 1993; Kubera et al., 2001; Serebruany et al., 2001). Additionally, SSRIs lower plasma 5-HT, reduce key aggregation glycoproteins, and inhibit platelet signaling proteins involved in calcium mobilization release, which is a key step in platelet activation (Maguire et al., 1993; Kubera et al., 2001; Serebruany et al., 2001; Malinin et al., 2004). In contrast to CNS neurons which can synthesize 5-HT *de novo* via tryptophan hydroxylase, an enzyme which has enhanced expression in response to SSRIs, platelets cannot synthesize 5-HT and experience a depletion of intracellular stores to less than 2% of baseline after SSRI treatment (Shah and Amini-Nik, 2017). Relevant for poorly perfusing wounds, preliminary animal model studies have shown SSRIs may increase endothelial nitric oxide synthase activity and nitric oxide bioavailability (Pereira et al., 2015; Ofek et al., 2012). The platelet-inhibitory SSRI effects may also be particularly useful for targeting venous stasis ulcer disease processes as all classes of chronic venous insufficiency are linked to pathogenic platelet hyperactivity and increased platelet-monocyte and platelet-neutrophil aggregates, independent of whether a wound is present (Malinin et al., 2004).

### 2.3 Inflammation phase immunomodulation

The downregulatory effects of SSRIs on platelet 5-HT signaling may also modulate the inflammatory stage of wound healing where platelet-secreted 5-HT enhances recruitment and activation of neutrophils and macrophages, leading to unfavorable upregulation of key pro-inflammatory cytokines including tumor necrosis factor alpha (TNF-ɑ) and interleukin-12 (IL-12) in chronic wounds (Maguire et al., 1993; Kubera et al., 2001; Serebruany et al., 2001; Shah and Amini-Nik, 2017). Synergistically, *in vitro* SSRI exposure has been shown to increase natural killer cell, a negative regulator of wound-microenvironment pro-inflammatory signaling, activity thereby reducing inflammation via two converging, complementary mechanisms (Frank et al., 1999; Brubaker et al., 2011). Dendritic cells are also involved in the inflammatory stage and sequester 5-HT via SERT from activated T lymphocytes, subsequently presenting it to naïve T cells to promote their activation and adaptive immune response (Shah and Amini-Nik, 2017). In pathologic nonhealing wounds, T lymphocytes are elevated and exhibit dysfunctional signaling unresponsive to stimulation (Raziyeva et al., 2021; Havran and Jameson, 2010; Shah and Amini-Nik, 2017). SSRIs may reduce T lymphocyte proliferation, cytokine production, and induce apoptosis in unresponsive, constitutively activated T lymphocytes preferentially compared to mature naïve T lymphocytes (Shenoy et al., 2013; Di Rosso et al., 2016; Pállinger and Csaba, 2007; Gobin et al., 2013). A persistent inflammatory phase may be associated with dysregulated monopoiesis and sustained elevation of proinflammatory macrophages, a key cell-type for transition to the proliferative stage which has upregulated phagocytosis and TNF-a in response to 5-HT (Li et al., 2021; Malinin et al., 2004). In an *ex-vivo* study, SSRIs significantly reduced the expression of CXCR4, CD4, and CCR5 on both monocyte-derived macrophages (MDM) and peripheral blood mononuclear cells compared to control, suggesting an inhibitory role of SSRIs for macrophage proinflammatory signaling (Greeson et al., 2016). Other studies have demonstrated SSRI-associated reductions in MDM proinflammatory cytokines, reactive oxygen species, and antigen presentation to immune cells, which may be dysregulated in pathologic wound healing (Nazimek et al., 2017).

### 2.4 Proliferation: keratinocyte and fibroblast responses

In regard to proliferative phase keratinocytes, topical 5-HT has been shown to enhance survival, migration, and wound area reduction in a dose-dependent manner, accelerating closure in both *in vitro* and *in vivo* models (Polanski et al., 1995; Sternberg et al., 1987; Nguyen et al., 2019; Sadiq et al., 2018; Lenz et al., 2001; Malinin et al., 2004; Seuwen et al., 1988; Rodriguez-Barucg et al., 2024). Improved keratinocyte scratch closure rates due to increased proliferation were reversed following treatment with ketanserin, a 5-HT receptor antagonist, supporting SSRI dependance on 5-HT keratinocyte signaling (Rodriguez-Barucg et al., 2024). This study also identified improved phosphorylation profiles and 350 differentially expressed genes in SSRI-treated keratinocytes with reactome analysis suggesting altered mitochondrial and ribonucleotide metabolism and thermogenesis (Rodriguez-Barucg et al., 2024). In fibroblasts, 5-HT promotes survival, activity, proliferation, and collagen production, while also synergizing with FGF-2 to enhance tissue proliferation (Polanski et al., 1995; Sternberg et al., 1987; Nguyen et al., 2019; Sadiq et al., 2018; Lenz et al., 2001; Malinin et al., 2004; Seuwen et al., 1988). These effects involve active transport, increased oxygen formation, protein phosphorylation, and 5-HT receptor–mediated mitogenesis, adhesion, and multiplication in culture (Polanski et al., 1995; Sternberg et al., 1987; Nguyen et al., 2019; Sadiq et al., 2018; Lenz et al., 2001; Malinin et al., 2004; Seuwen et al., 1988). The effects of SSRIs on certain chronic wound mechanism which have implicated 5-HT signaling remain unassessed; these include 5-HT-induced B-lymphocyte proliferation, afferent nerve ending stimulation and pain response and the potential of SSRIs to alter β-receptor function in wound cells, similar to SSRI-induced postsynaptic β-receptor downregulation in the brain (Malinin et al., 2004).

### 2.5 Selective serotonin reuptake inhibitors and wound microbiome

Recurrent or chronic infection is a known contributor to impaired wound healing and, in a retrospective analysis of 2963 patients, it was found that the predominant bacterial species in chronic wounds were *S. epidermidis*, *S. aureus*, *Corynebacterium*, and *Pseudomonadaceae* (Kiecka and Szczepanik, 2022). Biofilms, which hinder antibiotic treatment by using extracellular polymeric substance barriers and efflux pumps, are associated with delayed healing and increased infection risk in chronic wounds (Gompelman et al., 2016; Wall et al., 2019; Pereira et al., 2021). Polymicrobial interactions, involving species like *Staphylococcus aureus* and *Pseudomonas aeruginosa* may enhance biofilm pathogenicity via exchange of antibiotic resistance genes, like those producing antibiotic-degrading enzymes (Ponde et al., 2021; Cheong et al., 2021; Orazi and O'Toole, 2019).

Among SSRIs, fluoxetine and sertraline have the strongest antimicrobial effects; these SSRIs are more hydrophobic than others and may diffuse more easily across the phospholipid membrane to interact with cellular machinery (Kiecka and Szczepanik, 2022). These SSRIs, at sub-minimum inhibitory concentrations, have been shown to prevent biofilm production via ALS3 protein-binding and reduce mature biofilm metabolism (Oliveira et al., 2018; Costa Silva et al., 2017; Rodrigues et al., 2023). Fluoxetine has been shown to have *in vitro* effect against multi-resistant *S. aureus, E. faecalis, S. epidermidis, E. coli and P. aeruginosa* and decreases biofilm formation of *S. aureus* clinical isolate UAMS-1 (Dafinone et al., 2025). There is also evidence suggesting fluoxetine synergizes polymyxin B bactericidal effects in 80% of gram-negative isolates, outperforming fosfomycin and meropenem combinations (Ahmed et al., 2024). Fluoxetine topical application to infected wounds may also decrease purulence and hinder hematogenous bacterial invasion (Dafinone et al., 2025). Fluoxetine has also shown effectiveness against fluconazole-resistant *Candida* strains, and its administration leads to significant dose-dependent reductions in biofilm metabolism, 96% for *C. krusei,* and biomass, 82% for *C. glabrat*a (Costa Silva et al., 2017). Sertraline inhibits the growth of *S. aureus*, *E. coli*, *P. aeruginosa*, and other Gram-positive bacteria such as *S. epidermidis* and *E. faecalis*, and shows synergy with antibiotics, including reductions in mimimum inhibitory concentration of tetracycline and ciprofloxacin against *C. urealyticum* and quinolone-resistant strains (Dafinone et al., 2025). While generally less effective against Gram-negative species, it demonstrates activity against *H. influenzae, M. catarrhalis, C. jejuni, Acinetobacter* spp., and can inhibit biofilm production in coagulase-negative staphylococci (Dafinone et al., 2025). In addition to the immunomodulatory properties of SSRIs, the antimicrobial properties of SSRIs make them promising adjuncts for difficult-to-treat wound bacterial and fungal infection.

## 3 Topical selective serotonin reuptake inhibitors and wound healing studies

### 3.1 Pre-clinical studies

Evidence from preclinical studies suggest topical SSRI application may improve wound healing. An *in vivo* study of diabetic mouse wound models revealed topical fluoxetine treatment promoted re-epithelialization, with significant decreases in wound area and exudate. Topical fluoxetine treatment also increased angiogenesis, suggested by higher CD31^+^ endothelial cell counts and visible small vessels, while reducing inflammatory macrophages and shifting their phenotype towards a pro-reparative state (Nguyen et al., 2019). In another study where wounds were created in rats, chronic topical fluoxetine administration improved mean wound lengths compared to acute administration in the initial 4 days; wounds in both chronic and acute fluoxetine treatment groups healed completely by day 10, while the placebo group did not fully heal by the study’s conclusion (Farahani et al., 2007) In rats subjected to stress which decreased wound healing rate, topical fluoxetine treatment increased healing rate, leukocyte healing response, and normalized epithelialization and epithelial cell structure (Farahani et al., 2007). Bioelectronic delivery of topical fluoxetine in a murine punch wound model increased re-epithelialization by nearly 40% compared to control; this was accompanied by anti-inflammatory M2 macrophage infiltration without pro-inflammatory M1 presence, leading to a reduced M1/M2 ratio over time, indicative of accelerated transition to the reparative phase of wound healing (Li et al., 2024; Asefifeyzabadi et al., 2024). In a porcine model also using bioelectronic delivery, topical fluoxetine produced similar re-epithelialization improvements, also with corresponding improvements in the wound macrophage and cytokine profile (Li et al., 2025).There is an advantage to topical, as opposed to systemic, administration: preclinical data indicate that topical fluoxetine application leads to plasma fluoxetine concentrations that are twofold lower than those achieved with oral fluoxetine at therapeutic neurological doses. Importantly, topical fluoxetine treatment does not affect plasma levels of 5-HT, highlighting its potential for localized therapy with minimal systemic impact (Nguyen et al., 2019).

### 3.2 Clinical studies

In view of the pro-reparative outcomes noted with agonists of the 5-HT receptors, it may be counterintuitive to propose that antagonists may have a similar result. However, existing clinical studies have investigated ketanserin. Although ketanserin does not increase serotonin levels, its wound healing effects are thought to result from its antiplatelet properties and ability to improve microvascular perfusion, rather than through direct serotonergic signaling (Malinin et al., 2004; Hedner and Persson, 1988). Separately, iproniazid (an irreversible monoamine oxidase inhibitor that limits the breakdown of serotonin) may promote reparative effects by increasing extracellular 5-HT and enhancing downstream receptor activation (Gillman, 2005). There are no ongoing or previous clinical trials investigating topical SSRIs for the treatment of chronic wounds (ClinicalTrials.gov).

In a double-blind placebo-controlled clinical trial of various chronic wounds, topical ketanserin 2% BID application was associated with greater re-epithelialization, granulation tissue formation, and wound area reduction rate (Janssen et al., 1989). Other studies of topical ketanserin 2% BID for VLUs and DFUs showed similar improvements in wound healing measures in addition to reductions in transudate and erythema (Roelens, 1989). In a double-blind intra-individual comparative study, diabetic participants with ≥2 chronic leg ulcers were randomized to receive topical 2% ketanserin BID on one ulcer and placebo on another ulcer for 8 weeks, in adjunct to SOC. The mean weekly reduction in wound area was significantly greater for ketanserin-treated ulcers (10.25%) compared to placebo-treated ulcers (2.5%) (Quatresooz et al., 2006). One trial investigating topical 10% iproniazid BID for 2 weeks for the treatment of chronic ulcers showed significantly increased rates of healing throughout the trial compared to placebo control (Goldstein et al., 1962). These studies are summarized in Table 1.

**TABLE 1:** Clinical studies investigating topical serotonin-modulating medications and chronic wound healing.

Treatment	Participants (n)	Ulcer etiology	Findings	Reference
2% Ketanserin BID	72	VLU, Decubitus, or Ischemic	Ketanserin significantly improved granulation (p < 0.05), epithelialization, and reduced wound area faster than placebo, with 36% complete healing by week 8	Janssen et al. (1989)
2% Ketanserin BID	23	VLU	Ketanserin significantly improved granulation tissue formation compared to placebo (p < 0.05) and showed better epithelialization and healing (p < 0.01)	Roelens (1989)
2% Ketanserin BID	12	DFU	Ketanserin significantly reduced ulcer area by 94% (p < 0.001) and improved relative wound area and healing index values from week 4 onward (p < 0.05)	Quatresooz et al. (2006)
2% Ketanserin BID	140	DFU	At 12 weeks, ketanserin reduced ulcer area by 87% vs. 63% for placebo, significantly accelerating wound healing	Martínez-de Jesús et al. (1997)
10% Iproniazid BID	28	Decubitus or Traumatic	After 1 week, iproniazid-treated lesions healed 52% vs. 27% for saline (p < 0.05), and differences remained significant after 2 weeks	Goldstein et al. (1962)

## 4 Selective serotonin reuptake inhibitor-associated cutaneous adverse drug reactions

Oral SSRI-associated cutaneous adverse drug reactions (CADRs) have been reported in the literature. One systematic review of 173 cases found fluoxetine is the most commonly reported SSRI with CADRs, followed by sertraline and paroxetine. CADRs were frequently petechiae, ecchymoses, alopecia, and photo-dermatoses (Masuka et al., 2022). SSRI-related petechiae and ecchymoses may be attributed to SSRI-induced platelet inhibition; however, a study of oral fluoxetine found no significant difference in cutaneous microcirculation compared to control (Andrade et al., 2010; Edinoff et al., 2022; Mück-Weymann and Rechlin, 1996). Notably, there are no reports of adverse drug reactions for topical SSRI administration. Therefore, topical delivery of SSRIs for the treatment of chronic wounds or wound infections is preferred, to maximize concentrations of the drug at the wound while minimizing systemic absorption. Indeed, these approaches are under investigation, including delivery via hydrogels, microparticles, nano capsules, or bioelectronic delivery devices (Josino et al., 2021; Dos Santos et al., 2020). Topical use would be expected to minimize serotonergic effects; although, patients on dual antiplatelet therapy or anticoagulation should still be monitored clinically.

## 5 Conclusion

With their immunomodulatory and antimicrobial properties, repurposing 5-HT modulating pharmacotherapy, including SSRIs, for topical administration may offer significant benefits in treating chronic wounds, as supported by both preclinical and clinical evidence. Further clinical research and pharmacokinetic studies are essential to fully evaluate the potential of SSRIs in improving wound healing outcomes and to establish their role as a viable adjunctive therapeutic option in chronic wound care.

## References

[B1] Ahmed S. A. Jordan R. L. Isseroff R. R. Lenhard J. R. (2024). Potential synergy of fluoxetine and antibacterial agents against skin and soft tissue pathogens and drug-resistant organisms. Antibiot. (Basel) 13, 1165. 10.3390/antibiotics13121165 39766555 PMC11672584

[B2] Almalki S. G. Agrawal D. K. (2017). ERK signaling is required for VEGF-A/VEGFR2-induced differentiation of porcine adipose-derived mesenchymal stem cells into endothelial cells. Stem Cell Res. Ther. 8, 113. 10.1186/s13287-017-0568-4 28499402 PMC5429549

[B3] Alstergren P. Ernberg M. Kopp S. Lundeberg T. Theodorsson E. (1999). TMJ pain in relation to circulating neuropeptide Y, serotonin, and interleukin-1 beta in rheumatoid arthritis. J. Orofac. Pain 13, 49–55. 10425968

[B4] Andrade C. Sandarsh S. Chethan K. B. Nagesh K. S. (2010). Serotonin reuptake inhibitor antidepressants and abnormal bleeding: a review for clinicians and a reconsideration of mechanisms. J. Clin. Psychiatry 71, 1565–1575. 10.4088/JCP.09r05786blu 21190637

[B5] Asefifeyzabadi N. Nguyen T. Li H. Zhu K. Yang H. Y. Baniya P. (2024). A pro-reparative bioelectronic device for controlled delivery of ions and biomolecules. Wound Repair Regen. 32, 709–719. 10.1111/wrr.13191 38794912

[B6] Brody D. J. Gu Q. (2020). Antidepressant use among adults: united States, 2015-2018 *NCHS data brief* . MD: Hyattsville. code data-end=”628” data-start=”611. 33054926

[B7] Brubaker A. L. Schneider D. F. Kovacs E. J. (2011). Neutrophils and natural killer T cells as negative regulators of wound healing. Expert Rev. Dermatol 6, 5–8. 10.1586/edm.10.66 21442028 PMC3063646

[B8] Ceradini D. J. Kulkarni A. R. Callaghan M. J. Tepper O. M. Bastidas N. Kleinman M. E. (2004). Progenitor cell trafficking is regulated by hypoxic gradients through HIF-1 induction of SDF-1. Nat. Med. 10, 858–864. 10.1038/nm1075 15235597

[B9] Cheong J. Z. A. Johnson C. J. Wan H. Liu A. Kernien J. F. Gibson A. L. F. (2021). Priority effects dictate community structure and alter virulence of fungal-bacterial biofilms. ISME J. 15, 2012–2027. 10.1038/s41396-021-00901-5 33558690 PMC8245565

[B10] Costa Silva R. A. Da Silva C. R. De Andrade Neto J. B. Da Silva A. R. Campos R. S. Sampaio L. S. (2017). *In vitro* anti-candida activity of selective serotonin reuptake inhibitors against fluconazole-resistant strains and their activity against biofilm-forming isolates. Microb. Pathog. 107, 341–348. 10.1016/j.micpath.2017.04.008 28411060

[B11] Dafinone M. E. Lyle R. E. Lee C. Mehta A. Dahle S. E. Isseroff R. R. (2025). Non-antibiotic approaches to mitigating wound infections: potential for SSRIs and adrenergic antagonists as emerging therapeutics. Wound Repair Regen. 33, e13240. 10.1111/wrr.13240 39737521

[B12] Di Rosso M. E. Palumbo M. L. Genaro A. M. (2016). Immunomodulatory effects of fluoxetine: a new potential pharmacological action for a classic antidepressant drug? Pharmacol. Res. 109, 101–107. 10.1016/j.phrs.2015.11.021 26644208

[B13] Dos Santos S. B. F. Pereira S. A. Rodrigues F. A. M. Da Silva A. C. C. De Almeida R. R. Sousa A. C. C. (2020). Antibacterial activity of fluoxetine-loaded starch nanocapsules. Int. J. Biol. Macromol. 164, 2813–2817. 10.1016/j.ijbiomac.2020.08.184 32853612

[B14] Duerschmied D. Suidan G. L. Demers M. Herr N. Carbo C. Brill A. (2013). Platelet serotonin promotes the recruitment of neutrophils to sites of acute inflammation in mice. Blood 121, 1008–1015. 10.1182/blood-2012-06-437392 23243271 PMC3567335

[B15] Edinoff A. N. Raveendran K. Colon M. A. Thomas B. H. Trettin K. A. Hunt G. W. (2022). Selective serotonin reuptake inhibitors and associated bleeding risks: a narrative and clinical review. Health Psychol. Res. 10, 39580. 10.52965/001c.39580 36425234 PMC9680839

[B16] Enoch S. Leaper D. J. (2008). “Basic science of wound healing,”, 26. Oxford, 31–37. 10.1016/j.mpsur.2007.11.005 Surgery

[B17] Eskeland S. Halvorsen J. A. Tanum L. (2017). Antidepressants have anti-inflammatory effects that may be relevant to dermatology: a systematic review. Acta Derm. Venereol. 97, 897–905. 10.2340/00015555-2702 28512664

[B18] Farahani R. M. Sadr K. Rad J. S. Mesgari M. (2007). Fluoxetine enhances cutaneous wound healing in chronically stressed wistar rats. Adv. Skin. Wound Care 20, 157–165. 10.1097/01.ASW.0000262710.59293.6b 17473722

[B19] Flaumenhaft R. De Ceunynck K. (2017). Targeting PAR1: now what? Trends Pharmacol. Sci. 38, 701–716. 10.1016/j.tips.2017.05.001 28558960 PMC5580498

[B20] Frank M. G. Hendricks S. E. Johnson D. R. Wieseler J. L. Burke W. J. (1999). Antidepressants augment natural killer cell activity: *in vivo* and *in vitro* . Neuropsychobiology 39, 18–24. 10.1159/000026555 9892855

[B21] Gillman P. K. (2005). Monoamine oxidase inhibitors, opioid analgesics and serotonin toxicity. Br. J. Anaesth. 95, 434–441. 10.1093/bja/aei210 16051647

[B22] Gobin V. Van Steendam K. Fevery S. Tilleman K. Billiau A. D. Denys D. (2013). Fluoxetine reduces murine graft-versus-host disease by induction of t cell immunosuppression. J. Neuroimmune Pharmacol. 8, 934–943. 10.1007/s11481-013-9463-7 23640520 PMC3737435

[B23] Goldstein H. S. Wolcott M. W. Rosenstein I. N. (1962). Iproniazid in healing of chronic topical ulcers. Arch. Surg. 85, 1011–1015. 10.1001/archsurg.1962.01310060147026 13948807

[B24] Gompelman M. Van Asten S. A. V. Peters E. J. G. (2016). Update on the role of infection and biofilms in wound healing: pathophysiology and treatment. Plast. Reconstr. Surg. 138, 61S-70S–70S. 10.1097/PRS.0000000000002679 27556776

[B25] Gonzalez De Valdivia E. Broselid S. Kahn R. Olde B. Leeb-Lundberg L. M. F. (2017). G protein-coupled estrogen receptor 1 (GPER1)/GPR30 increases ERK1/2 activity through PDZ motif-dependent and -independent mechanisms. J. Biol. Chem. 292, 9932–9943. 10.1074/jbc.M116.765875 28450397 PMC5473245

[B26] Greeson J. M. Gettes D. R. Spitsin S. Dubé B. Benton T. D. Lynch K. G. (2016). The selective serotonin reuptake inhibitor citalopram decreases human immunodeficiency virus receptor and coreceptor expression in immune cells. Biol. Psychiatry 80, 33–39. 10.1016/j.biopsych.2015.11.003 26725193 PMC4862937

[B27] Guo P. Tai Y. Wang M. Sun H. Zhang L. Wei W. (2022). Gα12 and Gα13: versatility in physiology and pathology. Front. Cell Dev. Biol. 10, 809425. 10.3389/fcell.2022.809425 35237598 PMC8883321

[B28] Havran W. L. Jameson J. M. (2010). Epidermal T cells and wound healing. J. Immunol. 184, 5423–5428. 10.4049/jimmunol.0902733 20483798 PMC2944652

[B29] Hedner T. Persson B. (1988). Effects of a new serotonin antagonist, ketanserin, in experimental and clinical hypertension. Am. J. Hypertens. 1, 317S-323S–323S. 10.1093/ajh/1.3.317s 3046635

[B30] Huang Z. Miao X. Luan Y. Zhu L. Kong F. Lu Q. (2015). PAR1‐stimulated platelet releasate promotes angiogenic activities of endothelial progenitor cells more potently than PAR4‐stimulated platelet releasate. J. Thrombosis Haemostasis 13, 465–476. 10.1111/jth.12815 25495701

[B31] Iwabayashi M. Taniyama Y. Sanada F. Azuma J. Iekushi K. Kusunoki H. (2012). Role of serotonin in angiogenesis: induction of angiogenesis by sarpogrelate *via* endothelial 5-HT1B/Akt/eNOS pathway in diabetic mice. Atherosclerosis 220, 337–342. 10.1016/j.atherosclerosis.2011.10.042 22172591

[B32] Janssen P. A. J. Janssen H. Cauwenbergh G. Doncker P. D. Beule K. D. Lewi P. (1989). Use of topical ketanserin in the treatment of skin ulcers: a double-blind study. J. Am. Acad. Dermatology 21, 85–90. 10.1016/s0190-9622(89)70153-5 2663937

[B33] Josino M. A. A. Rocha Da Silva C. De Andrade Neto J. B. Barroso F. D. D. Juvêncio Da Silva L. Cavalcanti B. C. (2021). Development and *in vitro* evaluation of microparticles of fluoxetine in galactomannan against biofilms of S. aureus methicilin resistant. Carbohydr. Polym. 252, 117184. 10.1016/j.carbpol.2020.117184 33183631

[B34] Kiecka A. Szczepanik M. (2022). The potential action of SSRIs in the treatment of skin diseases including atopic dermatitis and slow-healing wounds. Pharmacol. Rep. 74, 947–955. 10.1007/s43440-022-00423-7 36203121 PMC9584846

[B35] Kubera M. Lin A. H. Kenis G. Bosmans E. Van Bockstaele D. Maes M. (2001). Anti-inflammatory effects of antidepressants through suppression of the interferon-gamma/interleukin-10 production ratio. J. Clin. Psychopharmacol. 21, 199–206. 10.1097/00004714-200104000-00012 11270917

[B36] Laberge S. Cruikshank W. W. Beer D. J. Center D. M. (1996). Secretion of IL-16 (lymphocyte chemoattractant factor) from serotonin-stimulated CD8+ T cells *in vitro* . J. Immunol. 156, 310–315. 10.4049/jimmunol.156.1.310 8598478

[B37] Lenz G. Gonçalves D. Luo Z. Avruch J. Rodnight R. Neary J. T. (2001). Extracellular ATP stimulates an inhibitory pathway towards growth factor-induced cRaf-1 and MEKK activation in astrocyte cultures. J. Neurochem. 77, 1001–1009. 10.1046/j.1471-4159.2001.00299.x 11359865

[B38] Li M. Hou Q. Zhong L. Zhao Y. Fu X. (2021). Macrophage related chronic inflammation in non-healing wounds. Front. Immunol. 12, 681710. 10.3389/fimmu.2021.681710 34220830 PMC8242337

[B39] Li H. Yang H.-Y. Asefifeyzabadi N. Baniya P. Lopez A. M. Gallegos A. (2024). Programmable delivery of fluoxetine via wearable bioelectronics for wound healing *in vivo* . Adv. Mater. Technol. 9, 2301115. 10.1002/admt.202301115

[B40] Li H. Asefifeyzabadi N. Schorger K. Baniya P. Yang H.-Y. Tebyani M. (2025). Remote-controlled wireless bioelectronics for fluoxetine therapy to promote wound healing in a porcine model. Adv. Mater. Technol. 10, 70039. n/a. 10.1002/admt.202500040

[B41] Lin P. K. Koller G. M. Davis G. E. (2023). Elucidating the morphogenic and signaling roles of defined growth factors controlling human endothelial cell lumen formation *versus* sprouting behavior. Am. J. Pathol. 193, 2203–2217. 10.1016/j.ajpath.2023.08.009 37689384 PMC10699133

[B42] Machida T. Iizuka K. Hirafuji M. (2013). 5-hydroxytryptamine and its receptors in systemic vascular walls. Biol. Pharm. Bull. 36, 1416–1419. 10.1248/bpb.b13-00344 23995652

[B43] Maguire K. Tuckwell V. Pereira A. Dean B. Singh B. (1993). Significant correlation between 14C-5-HT uptake by and 3H-paroxetine binding to platelets from healthy volunteers. Biol. Psychiatry 34, 356–360. 10.1016/0006-3223(93)90179-h 8218602

[B44] Malinin A. Oshrine B. Serebruany V. (2004). Treatment with selective serotonin reuptake inhibitors for enhancing wound healing. Med. Hypotheses 63, 103–109. 10.1016/j.mehy.2003.10.021 15193359

[B45] Martínez-De Jesús F. R. Morales-Guzmán M. Castañeda M. Pérez-Morales A. García-Alonso J. Mendiola-Segura I. (1997). Randomized single-blind trial of topical ketanserin for healing acceleration of diabetic foot ulcers. Arch. Med. Res. 28, 95–99. 9078595

[B46] Masuka J. T. Mchunu N. Mkhize Z. Thandar Y. Mosam A. (2022). Selective serotonin reuptake inhibitor and serotonin-norepinephrine reuptake inhibitor associated cutaneous adverse drug reactions: a systematic review of case reports and case series. Australas. J. Dermatol 63, e13–e20. 10.1111/ajd.13780 34958129

[B47] Mück-Weymann M. Rechlin T. (1996). Reflexes of the cutaneous microcirculation in amitriptyline and in fluoxetine treated patients. Psychopharmacol. Berl. 124, 241–244. 10.1007/BF02246663 8740045

[B48] Nazimek K. Strobel S. Bryniarski P. Kozlowski M. Filipczak-Bryniarska I. Bryniarski K. (2017). The role of macrophages in anti-inflammatory activity of antidepressant drugs. Immunobiology 222, 823–830. 10.1016/j.imbio.2016.07.001 27453459

[B49] Nguyen C. M. Tartar D. M. Bagood M. D. So M. Nguyen A. V. Gallegos A. (2019). Topical fluoxetine as a novel therapeutic that improves wound healing in diabetic mice. Diabetes 68, 1499–1507. 10.2337/db18-1146 31048368 PMC6609984

[B50] Ofek K. Schoknecht K. Melamed-Book N. Heinemann U. Friedman A. Soreq H. (2012). Fluoxetine induces vasodilatation of cerebral arterioles by co-modulating NO/muscarinic signalling. J. Cell Mol. Med. 16, 2736–2744. 10.1111/j.1582-4934.2012.01596.x 22697296 PMC4118242

[B51] Oliveira A. S. Martinez-De-Oliveira J. Donders G. G. G. Palmeira-De-Oliveira R. Palmeira-De-Oliveira A. (2018). Anti-candida activity of antidepressants sertraline and fluoxetine: effect upon pre-formed biofilms. Med. Microbiol. Immunol. 207, 195–200. 10.1007/s00430-018-0539-0 29556778

[B78] Opneja A. Kapoor S. Stavrou E. X. (2019). Contribution of platelets, the coagulation and fibrinolytic systems to cutaneous wound healing. Thromb. Res. 179, 56–63. 10.1016/j.thromres.2019.05.001 31078121 PMC6556139

[B52] Olszewska-Pazdrak B. Carney D. H. (2013). Systemic administration of thrombin peptide TP508 enhances VEGF-stimulated angiogenesis and attenuates effects of chronic hypoxia. J. Vasc. Res. 50, 186–196. 10.1159/000348250 23594718 PMC4603662

[B53] Orazi G. O'toole G. A. (2019). It takes A village: mechanisms underlying antimicrobial recalcitrance of polymicrobial biofilms. J. Bacteriol. 202, e00530-19. 10.1128/JB.00530-19 31548277 PMC6932244

[B54] Pállinger E. Csaba G. (2007). Effect of serotonin-acting agents on the serotonin content of immune cells. A peculiar observation. Cell Biochem. Funct. 25, 581–583. 10.1002/cbf.1329 16615040

[B55] Pereira C. A. Ferreira N. S. Mestriner F. L. Antunes-Rodrigues J. Evora P. R. Resstel L. B. (2015). Chronic fluoxetine treatment increases NO bioavailability and calcium-sensitive potassium channels activation in rat mesenteric resistance arteries. Eur. J. Pharmacol. 765, 375–383. 10.1016/j.ejphar.2015.09.002 26362752

[B56] Pereira R. Dos Santos Fontenelle R. O. De Brito E. H. S. De Morais S. M. (2021). Biofilm of candida albicans: formation, regulation and resistance. J. Appl. Microbiol. 131, 11–22. 10.1111/jam.14949 33249681

[B57] Polanski M. Vermeulen M. W. Wu J. Karnovsky M. L. (1995). Muramyl dipeptide mimicry in the regulation of murine macrophage activation by serotonin. Int. J. Immunopharmacol. 17, 225–232. 10.1016/0192-0561(94)00097-8 7558518

[B58] Ponde N. O. Lortal L. Ramage G. Naglik J. R. Richardson J. P. (2021). *Candida albicans* biofilms and polymicrobial interactions. Crit. Rev. Microbiol. 47, 91–111. 10.1080/1040841X.2020.1843400 33482069 PMC7903066

[B59] Qin L. Zhao D. Xu J. Ren X. Terwilliger E. F. Parangi S. (2013). The vascular permeabilizing factors histamine and serotonin induce angiogenesis through TR3/Nur77 and subsequently truncate it through Thrombospondin-1. Blood 121, 2154–2164. 10.1182/blood-2012-07-443903 23315169 PMC3596973

[B60] Quatresooz P. Kharfi M. Paquet P. Vroome V. Cauwenbergh G. Piérard G. E. (2006). Healing effect of ketanserin on chronic leg ulcers in patients with diabetes. J. Eur. Acad. Dermatology Venereol. 20, 277–281. 10.1111/j.1468-3083.2006.01422.x 16503887

[B61] Raote I. Bhattacharya A. Panicker M. M. (2007). “Serotonin 2A (5-HT2A) receptor function: ligand-dependent mechanisms and pathways,” in Serotonin receptors in neurobiology. Editor Amitabha C. (Boca Raton, FL: Taylor & Francis). 21204452

[B62] Raziyeva K. Kim Y. Zharkinbekov Z. Kassymbek K. Jimi S. Saparov A. (2021). Immunology of acute and chronic wound healing. Biomolecules 11, 700. 10.3390/biom11050700 34066746 PMC8150999

[B63] Rodrigues D. S. Cabral V. P. F. Barbosa A. D. Sá L. G. D. A. Moreira L. E. A. De Andrade Neto J. B. (2023). Sertraline has *in vitro* activity against both mature and forming biofilms of different *candida* species. J. Med. Microbiol. 72. 10.1099/jmm.0.001664 36762524

[B64] Rodriguez-Barucg Q. Garcia A. A. Garcia-Merino B. Akinmola T. Okotie-Eboh T. Francis T. (2024). Environmental fluoxetine promotes skin cell proliferation and wound healing. Environ. Pollut. 362, 124952. 10.1016/j.envpol.2024.124952 39277126

[B65] Roelens P. (1989). Double-blind placebo-controlled study with topical 2% ketanserin ointment in the treatment of venous ulcers. Dermatologica 178, 98–102. 10.1159/000248400 2647535

[B66] Sadiq A. Shah A. Jeschke M. G. Belo C. Qasim Hayat M. Murad S. (2018). The role of serotonin during skin healing in post-thermal injury. Int. J. Mol. Sci. 19, 1034. 10.3390/ijms19041034 29596386 PMC5979562

[B67] Sen C. K. (2019). Human wounds and its burden: an updated compendium of estimates. Adv. Wound Care 8, 39–48. 10.1089/wound.2019.0946 30809421 PMC6389759

[B68] Sen C. K. (2021). Human wound and its burden: updated 2020 compendium of estimates. Adv. Wound Care (New Rochelle) 10, 281–292. 10.1089/wound.2021.0026 33733885 PMC8024242

[B69] Serebruany V. L. Gurbel P. A. O'connor C. M. (2001). Platelet inhibition by sertraline and N-desmethylsertraline: a possible missing link between depression, coronary events, and mortality benefits of selective serotonin reuptake inhibitors. Pharmacol. Res. 43, 453–462. 10.1006/phrs.2001.0817 11394937

[B70] Seuwen K. Magnaldo I. Pouysségur J. (1988). Serotonin stimulates DNA synthesis in fibroblasts acting through 5-HT1B receptors coupled to a Gi-protein. Nature 335, 254–256. 10.1038/335254a0 3045568

[B71] Shah A. Amini-Nik S. (2017). The role of serotoninergic system in skin healing. Int. J. Drug Res. Technol. 7, 80–106.

[B72] Shenoy A. R. Dehmel T. Stettner M. Kremer D. Kieseier B. C. Hartung H. P. (2013). Citalopram suppresses thymocyte cytokine production. J. Neuroimmunol. 262, 46–52. 10.1016/j.jneuroim.2013.06.006 23886473

[B73] Sternberg E. M. Wedner H. J. Leung M. K. Parker C. W. (1987). Effect of serotonin (5-HT) and other monoamines on murine macrophages: modulation of interferon-gamma induced phagocytosis. J. Immunol. 138, 4360–4365. 10.4049/jimmunol.138.12.4360 3108386

[B74] Tsopanoglou N. E. Maragoudakis M. E. (1999). On the mechanism of thrombin-induced angiogenesis. Potentiation of vascular endothelial growth factor activity on endothelial cells by up-regulation of its receptors. J. Biol. Chem. 274, 23969–23976. 10.1074/jbc.274.34.23969 10446165

[B75] Wall G. Montelongo-Jauregui D. Vidal Bonifacio B. Lopez-Ribot J. L. Uppuluri P. (2019). Candida albicans biofilm growth and dispersal: contributions to pathogenesis. Curr. Opin. Microbiol. 52, 1–6. 10.1016/j.mib.2019.04.001 31085405 PMC6842673

[B76] Yang S. X. Chen J. H. Jiang X. F. Wang Q. L. Chen Z. Q. Zhao W. (2005). Activation of chemokine receptor CXCR4 in malignant glioma cells promotes the production of vascular endothelial growth factor. Biochem. Biophys. Res. Commun. 335, 523–528. 10.1016/j.bbrc.2005.07.113 16084492

[B77] Zhao R. Liang H. Clarke E. Jackson C. Xue M. (2016). Inflammation in chronic wounds. Int. J. Mol. Sci. 17, 2085. 10.3390/ijms17122085 27973441 PMC5187885

